# Scientific Production on Chemical Burns: A Bibliometric Analysis (1946–2024)

**DOI:** 10.3390/ebj6030051

**Published:** 2025-09-09

**Authors:** José-Enrique Cueva-Ramírez, Gregorio Gonzalez-Alcaide, Isabel Belinchón-Romero, Jose-Manuel Ramos-Rincon

**Affiliations:** 1 School of Morphological Sciences, Faculty of Health Sciences, Autonomous University of Santo Domingo, Santo Domingo 10105, Dominican Republic; 2 Pearl F. Ort Burn Unit, Santo Domingo 87052, Dominican Republic; 3 Department of History of Science and Documentation, University of Valencia, 46010 Valencia, Spain; gregorio.gonzalez@uv.es; 4 Department of Clinical Medicine, Miguel Hernández University of Elche, 03202 Elche, Spain; belinchon_isa@gva.es (I.B.-R.); jose.ramosr@umh.es (J.-M.R.-R.); 5 Service of Department, Dr. Balmis General University Hospital, 03010 Alicante, Spain; 6 Institute for Health and Biomedical Research (ISABIAL), 03010 Alicante, Spain; 7 Department of Internal Medicine, Dr. Balmis General University Hospital, 03010 Alicante, Spain

**Keywords:** burns, chemical burns, eyes burns, injury, management

## Abstract

Background: Chemical burns represent a persistent global health challenge due to their high prevalence, causing lifelong disabilities and socioeconomic burdens. Although research on chemical burns has expanded over the past century, no comprehensive study has mapped the intellectual structure, global collaboration patterns, and thematic evolution of scientific production on chemical burns to determine how research in the area has evolved and the existence of gaps or imbalances that need to be addressed. Objective: The aim was to analyze the scientific production on chemical burns using bibliometric methods, identifying key contributors, evolving themes, and research gaps. Methods: Eligible documents contained the MeSH descriptor and were listed both in PubMed (1946 to 2024) and in the Web of Science Core Collection. The documents were analyzed with Bibliometrix version 5.0 and VOSviewer version 1.6.20. The metrics included were annual productivity, citation networks, co-authorship patterns, and keyword co-occurrence. Results: The analysis included 3943 articles from 757 journals. The annual average was 25.8 articles, with a growth rate of 0.65% from 1946 to 2024. The USA produced the most articles (n = 1547), followed by China (n = 890). The USA also led in international collaboration, working with 26 countries. Harvard University was the leading institution (n = 325) and *Burns* the leading journal (n = 306), followed by Cornea (n = 132). The most common subject category of the research was surgery (n = 1185 docs) and ophthalmology (n = 984). Reim M. was the most prolific author (n = 35), while Basu S. had the most citations (n = 1159). The main clinical MeSH descriptors were “Eye burns” (n = 1158), “Esophageal stenosis” (n = 683), and “Caustics” (n = 659). Conclusions: The results show slight growth in scientific production on chemical burns. The USA and China are leading research in this field, and the main reported finding was eye burns.

## 1. Introduction

Chemical burns, resulting from exposure to corrosive agents such as acids, alkalis, and solvents, represent a critical public health challenge due to their potential to cause irreversible tissue damage, functional disability, and prolonged socioeconomic consequences [1]. Globally, an estimated 5% to 15% of all burn injuries are chemical in origin, with an annual incidence of up to 13 cases per 100,000 inhabitants in industrialized nations [2]. These injuries frequently occur in occupational settings (industrial chemical handling), domestic accidents (exposure to cleaning agents), and interpersonal violence, disproportionately affecting low-income regions where inadequate safety regulations and limited access to specialized care worsen clinical outcomes [3].

Pathophysiologically, chemical burns differ from thermal burns: alkalis (pH > 7) cause tissue liquefaction and deep penetration, whereas acids (pH < 7) induce coagulation and superficial eschar formation [4]. This heterogeneity, combined with variables such as agent concentration and exposure time, complicates therapeutic standardization.

For ocular burns—representing some of the most visually devastating injuries, the corneal stroma’s unique vulnerability to hypotonic solutions creates a paradoxical risk: while immediate irrigation is critical, standard water (hypotonic) may exacerbate damage by promoting osmotic influx of corrosive agents into deeper ocular structures [5]. This is particularly critical in pediatric cases, where thinner corneal epithelia and higher agent–surface area ratios accelerate penetration.

Current controversies center on three key challenges: (1) the optimal irrigation fluid osmolarity to balance emergency decontamination with stromal protection [6], (2) the risk–benefit profile of amphoteric solutions (e.g., Diphoterine) in children, whose developing ocular surface may show altered healing responses, and (3) the timing of surgical interventions like tenonplasty for severe burns to prevent anterior segment necrosis while minimizing inflammatory sequelae [6].

Recent advances include bioactive hydrogels for wound regeneration [7] and ex vivo models that quantify corrosive penetration kinetics (e.g., hydrofluoric acid [HF]’s 5 min full-thickness damage) [8], enabling targeted therapies. The ClinicalTrials.gov registry reveals a geographic imbalance: most chemical burn studies (2000–2025) are affiliated with HICs [9], raising concerns about generalizability to settings lacking specialized burn centers or costly therapies. Furthermore, existing systematic reviews have addressed narrow outcomes (e.g., objective scar assessment tools [10]), yet no study has comprehensively analyzed historical trends, collaboration networks, or emerging priorities in this field using bibliometrics.

This gap is particularly salient given that bibliometric analyses have proven valuable for mapping research patterns in other burn subtypes, such as electrical burns [11] and inhalation injuries [12]. These studies identified thematic trends, international collaborations, and critical knowledge gaps—providing a methodological framework applicable to chemical burns, where fragmented literature and geographic disparities [9] demand rigorous quantitative synthesis.

Given the complexity and multifaceted impact of chemical burns, a bibliometric approach is essential to evaluate the current state of scientific production. While broader burn injury research has been extensively documented [3,4,5,6,7,8,9], no prior study has systematically examined chemical burns through this lens.

In this specialized, multidisciplinary field, bibliometric analysis can strengthen research cohesion by identifying leading researchers, institutions, and countries, highlighting seminal publications and thematic clusters, and revealing underexplored areas in prevention, treatment, and outcomes [13]. To address these needs, this study aims to (1) trace the evolution of chemical burn research, (2) map landmark publications and knowledge domains, (3) analyze collaborative networks and geographic contributions, and (4) highlight translational gaps for future investigation.

## 2. Materials and Methods

### 2.1. Conceptual Definition of Study Area

To guarantee precision and standardization in identifying pertinent literature, we utilized the Medical Subject Headings (MeSH) database, curated by the US National Library of Medicine (NLM). Our search strategy incorporated the descriptor ‘Burns, Chemical’, defined by the NLM as tissue injuries resulting from exposure to acidic or alkaline agents.

### 2.2. Identification of Document Population

Two searches were conducted in the MEDLINE database and through the Web of Science platform, with the search string “Burns, Chemical” [Mesh]. No chronological limitations were established in the search process, although it should be noted that the coverage of documents in MEDLINE was focused from the mid-twentieth century to the present. Two experts in documentation and bibliometric studies designed the search strategy and undertook the analysis of the bibliographic information, advised by the other two authors participating in this study, who were specialists in the thematic area analyzed and who also participated in the interpretation of the results. The next step involved searching for the articles retrieved from MEDLINE in the Web of Science-Core Collection (WOS-CC) using PubMed IDs. By restricting the analysis to documents listed in WOS-CC, the analysis focused on the publications with the largest international circulation, and it was possible to determine the number of citations of the documents and all institutional affiliations of the authors over the period analyzed. Of a total of 6871 documents retrieved from MEDLINE, 4291 were also included in WOS-CC (62.45%).

Subsequently, documentary typologies that are not usually considered in bibliometric studies were excluded (n = 348). In total, 3943 documents were included in the bibliometric analysis after the exclusion of ineligible document types: editorials (n = 202), proceeding papers (n = 121), and other document types (n = 25). The chosen documents were the articles, reviews, and letters, from which the following information was extracted: title, journal, type of article, author names, affiliations, MeSH terms, publication date, research area, and abstract.

**Figure 1:**
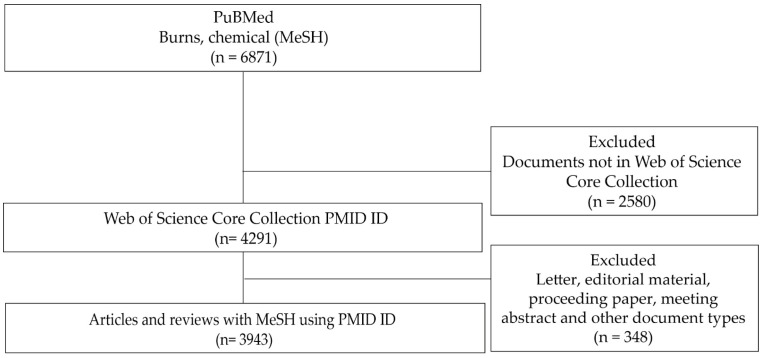
Flow chart of selection of documents on chemical burns.

Searches were conducted on 15 March 2025. Figure 1 illustrates the study selection process in a flow chart.

### 2.3. Indicators Obtained

The bibliometric analysis was based on the following indicators: number of documents published each year, number of documents by country, number of document citations by country, number of collaborations by country, number of documents by institution, number of documents by journal and by Journal Citation Reports (JCR) category, authors with the most documents, authors with the most citations, most-cited documents, and frequency distribution of MeSH terms assigned to documents.

### 2.4. Bibliometric Analysis and Visualization

The application selected to visualize collaborative networks of highly productive authors, to visualize publication production between countries, and to map international collaborations between countries was the Bibliometrix R package, version 5.0 (accessed via the Biblioshiny interface) [14].

In addition, the 2023 JCR impact factor was extracted as a measure of the influence and quality of scientific production. The categories of journals with publications were proportionally assigned in the case of multi-assigned journals: 2 categories = 0.5, 3 categories = 0.33, and 4 categories = 0.25. A co-occurrence network of MESH terms was generated to explore the interrelationships established between them. The Pajek 6.01 software tool was used for the graphical representations of MESH descriptors and the network based on thematic categories of the journals in which papers were published [15]. The MESH terms humans/animals, referring to sex (female and male), age groups, and study types were specifically analyzed.

The conception, planning of this study, data collection process, and data analysis were carried out by the authors responsible for this study. Only AI (ChatGPT version 3.5) was used to review and improve the writing and presentation of the contents.

## 3. Results

### 3.1. Main Information

The analysis of 757 journals and 3943 documents published from 1946 to 2024 revealed an annual growth rate of 0.65%. Original research articles were the most common document type (82.27%), followed by letters to the editor (10.25%) and review articles (4.44%). The database used for the analysis did not subclassify review types (systematic, narrative, etc.). Table S1 shows the main results of scientific production on chemical burns.

### 3.2. Publication Trends

**Figure 2:**
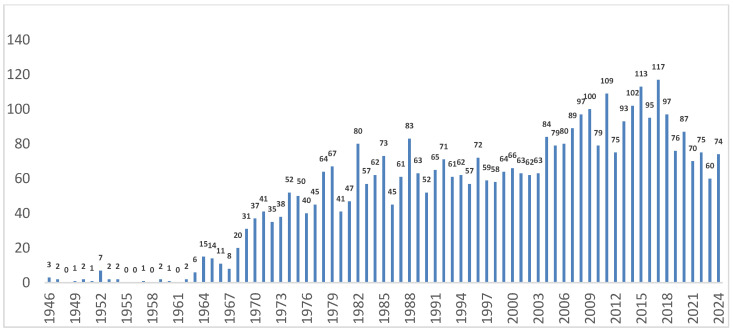
Annual scientific production on chemical burns (1946–2024).

There were 3943 articles published between 1946 and 2024; scientific production was low from 1946 to 1963. From 1964 onward, production began to increase gradually; the years with the highest scientific output were 2017 (117 articles), 2015 (113 articles), and 2011 (109 articles). Figure 2 displays the annual scientific production on chemical burns from 1946 to the present, while Figure S1 presents the scientific production divided into three periods (1946–1974, 1975–1999, and 2000–2024).

### 3.3. Country Analysis

The 3943 analyzed documents originated from 88 countries, with an uneven regional distribution: Europe contributed the highest number of countries (32), followed by Asia (25), Africa (16), the Americas (13), and Oceania (2). Table 1 details productivity metrics for the top 10 most prolific nations.

The top five corresponding author countries were the United States of America (USA) (n = 790), China (n = 323), the United Kingdom (UK) (n = 261), Turkey (n = 199), and India (n = 175). Regarding total citations and average citations per article, the order was Saudi Arabia (with 519 total citations [cits] and an average of 37.10 cits/document [doc]), Italy (2737 cits, with an average of 34.20 cits/docs), Switzerland (566 cits and 28.30 cits/docs), Finland (333 cits; 27.80 cits/docs), and Japan (2623 cits and 25.50 cits/docs. The USA, despite having the highest number of citations (n = 18,534), ranked seventh in average citations, with 23.50 cits/doc. Table S2 presents the top 30 most-cited countries and average cits/docs.

### 3.4. International Collaboration Between Countries

Among the top five countries in international collaboration, the USA led with 169 international collaborations with 44 countries, the UK with 59 collaborations with 35 countries, France with 42 collaborations with 25 countries, China with 31 collaborations with 13 countries, and Spain with 25 collaborations with 14 countries (Table S3).

### 3.5. Journal Analysis

Of the 697 journals that have published articles on chemical burns, the top 5 are *Burns* (n = 306), Cornea (n = 132), Investigative Ophthalmology & Visual Science (n = 99), Journal of Burn Care & Research (n = 75), and Journal of Pediatric Surgery (n = 68). Table 2 shows the category, JCR impact factor, and journal citation indicator (JCI) (2023 edition of JCR), year of first publication, and research areas of each journal in the field of chemical burns.

**Figure 3:**
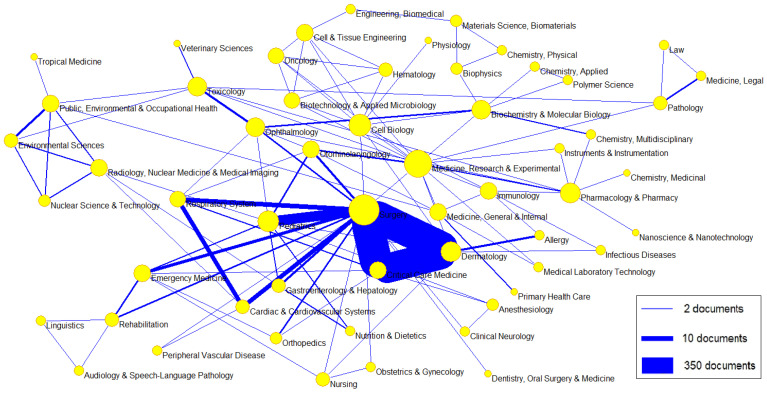
Network of links between the subject categories of scientific journals on chemical burns.

The most frequent category was Surgery (n = 1185 with an “N doc-weighted” value of 719.83); followed by Ophthalmology (n = 984 with an “N doc-weighted” value of 943; Dermatology (n = 547 with an “N doc-weighted” value of 260.7); Critical Care Medicine (n = 430, with an “N doc-weighted” value of 151); and Medicine, General and Internal (n = 388, with an “N doc-weighted” value of 375.17). Table 3 shows the number of documents assigned to the top 30 categories. Figure 3 illustrates the network of links between the subject categories of scientific journals in which the papers have been published. The main network is Surgery–Dermatology–Critical Care Medicine.

### 3.6. Author Analysis

The total number of authors who contributed to the production of these articles was 11,972; among them, there were 440 single-document authors, with an average number of authors per document of 4.04. The authors with the highest number of investigations were Reim M. (n = 35; h-index = 17; total citations [TC] = 694), Sarfati E. (n = 35; h-index = 16; TC = 836), Celerier M. (n = 32; h-index = 10; TC = 493), Pfister R.R. (n = 30; h-index = 19; TC = 936); Cattan P. (n = 23; h-index = 15; TC = 687), and Paterson C.A. (n = 22; h-index = 15; TC = 800). Table 4 also includes the total citations, the year of their first publication, and the topics covered by the authors.

The authors with the highest number of local citations were Pfister R.R. (n = 577); Paterson C.A. (n = 379), Reim M. (n = 370), Safarti E. (n = 361), and Kochhar R. (n = 302). Table S4 presents the list of the most relevant authors with the highest number of local citations.

### 3.7. Analysis of Most-Cited Articles

The article with the highest number of global citations (n = 1100) was written by Pellegrini G et al. in 1997, entitled “Long-Term Restoration of Damaged Corneal Surfaces with Autologous Cultivated Corneal Epithelium” [25] (with TC/year = 37.93), followed by Rama P et al. in 2010, who published “Limbal Stem-Cell Therapy and Long-Term Corneal Regeneration” [26] (TC = 884; TC/year = 55.25). Table 5 lists the top 10 articles with different citation metrics, the document type and category, and the research topic.

### 3.8. Analysis of MeSH Terms and Most Frequent Words

According to the weight identified by the frequency of MeSH terms, the main terms in the literature on chemical burns focus on eye burns, with 29.47% (n = 1158); esophageal stenosis, with 17.38% (n = 683); and caustics, with 16.77% (n = 659). These terms are the most recurrent and with the highest concentration of research. Other important terms, although with slightly less weight, include esophagus, with 13.51% (n = 659), and cornea, with 11.28% (n = 443), also indicating significant interest in these anatomical locations affected by chemical burns. Table 6 shows the top 30 MeSH terms distributed during the three periods (1946–1974, 1975–1999, and 2000–2024). Table S5 presents the top 60 MeSH terms.

Research on humans (n = 3197; 81.08% of the documents) was significantly ahead of research on animals (n = 933; 23.66%). In relation to sex, men were the subject of study in 53.89% (n = 2125) of the documents, compared with only 39.92% for women (n = 1574). Among age groups, adults predominated (n = 1339) compared with adolescents (n = 640), children (n = 610), and infants (n = 397). In relation to the types of studies, the two main categories, which stood out far above the rest, were “Retrospective Studies” (n = 386) and “Follow-Up Studies” (n = 275). Moreover, the most prominent topic from the WOS database was burns and damage by chemical agents, represented by words such as chemical burns, corrosive ingestion, toxic exposure, and injury, as shown in Figure S2.

### 3.9. Analysis of Thematic MeSH Terms

**Figure 4:**
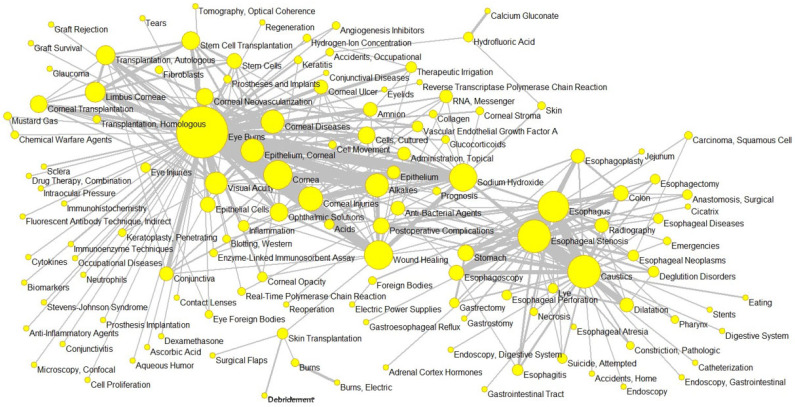
Medical subject heading (MeSH) thematic network in the scientific production on chemical burns (by Pajek).

Figure 4 shows the network of thematic MeSH terms. These relationships become evident through the thematic groups identified in the analysis. An important thematic MeSH term group topic was related to ophthalmology, directly linking the ocular burn node with cornea and other concepts, such as visual acuity. This reflects a substantial part of the research dedicated to chemical eye injuries and their consequences. Another significant thematic group of MeSH terms focused on gastroenterology, connecting the esophagus with esophageal stenosis, denoting a considerable body of work on chemical burns affecting the esophagus and their long-term complications. The caustics node acted as a fundamental connector, as these agents were the main cause of many of the ocular and esophageal burns investigated.

Additionally, an indirect connection was observed between the cornea node and the cell regeneration and transplants group, where therapies such as stem cell transplantation for the repair of corneal damage caused by burns were investigated. Although there was no thematic group that directly linked all the main nodes, the general concept of “burns” acted as an implicit central node that united them, representing the general context of chemical injuries and their diverse manifestations and causal agents studied in the literature.

### 3.10. Institutional Analysis

A total of 2320 institutions participated in the creation of the 3943 articles on chemical burns. Among those with the most publications on the topic stand out were Harvard University (n = 132) and Harvard Medical School (n = 85), totaling 217 publications between them; Universite Paris Cite (n = 121); Assistance Publique-Hôpitaux de Paris (n = 118); the L.V. Prasad Eye Institute (n = 91); and, in fifth place, the University of California System (n = 78). These universities belong to the USA, France, and India. Table 7 shows the 10 institutions’ production ranking, overall ranking, research ranking, and societal and social network ranking, as well as their line of research.

## 4. Discussion

This study provides us with an overview of research in this field over several decades. We observed that scientific production was initially low, initially associated with reductionist approaches to molecular pathophysiology studies [1], beginning a gradual increase from 1964 onward, and reaching notable peaks in the 2000s and 2010s. This progress reflected significant advances in two key areas.

In initial clinical management, the pioneering research of Pfister (1985) [18] and Kuckelkorn (2002) [6] established protocols for immediate irrigation and use of amphoteric solutions (such as Diphoterine), reducing complications such as corneal ulcerations. These findings support the surge in publications in the 2000s, where 34.5% of the studies analyzed focused on decontamination and first aid [35].

Another area focused on “regenerative therapies”, where the most-cited article (Pellegrini, 1997; TC = 1100) demonstrated that autologous limbal cell transplantation could restore damaged corneal surfaces [25], a milestone that drove 117 publications in 2017, many focused on bioengineering [7]. However, despite the relevance of this seminal paper on corneal epithelium restoration (TC/year = 37.93), citation metrics may be influenced by the journal’s high visibility (*The Lancet*) and the article’s early date. Notably, Rama et al. (2010) [26], though with fewer total citations (TC = 884), exhibited a higher annual citation rate (TC/year = 55.25), suggesting growing relevance in stem-cell applications.

The surge in regenerative therapy publications (e.g., the 117 studies in 2017) emerged from the convergence of Pellegrini’s seminal discovery (1997) [25]—which proved limbal stem cells from 1 mm^2^ biopsies could regenerate corneas—and subsequent advancements. By 2015, the industry had translated these findings into commercial therapies (e.g., EMA-approved Holoclar^®^), while bioengineering innovations, like fibrin scaffolds and 3D bioprinting, enabled more precise applications [7]. This synergy between basic science, clinical innovation, and technology fueled the publication peak.

Regarding the geographical distribution of research on the topic, the United States significantly leads in the number of publications (n = 1547). China, Turkey, the United Kingdom, and India are also among the top-producing countries.

It is interesting to note that while the USA has the highest total citations, together with China and Turkey, it accounts for 45% of publications. This contrasts with the high specialization of Italy and Saudi Arabia, which surpass them in citations per article, with 34.2 and 37.1, respectively. These differences respond to key factors that indicate differences in research focus or impact between these countries. Among these differences are the following:

- Regarding research and development, the USA and China concentrate 62% of clinical trials on advanced therapies (Rama, 2010) [26], while Turkey stands out in epidemiological studies on occupational burns [35].
- Regarding epidemiological contexts, in Bangladesh 68% of chemical burns are sulfuric acid attacks, a topic underrepresented in the global literature (only 2% of studies according to Mannan et al. [36]). This reflects the need to adapt research to local realities, as proposed by Forjuoh for low-income countries [2].
- Regarding technological access, while Europe and the US dominate corneal transplant research [20], Africa (16 countries, and 1.2% of publications) shows a critical gap in primary prevention studies, particularly for pediatric burns, which have high incidence rates in the region [37].

Among international collaborations, the collaborations between Harvard and Université Paris Cité (15% of joint publications) have driven combined therapies (e.g., bioengineering and anti-inflammatories) as key to reducing corneal fibrosis [27].

China mainly collaborates with Asian countries (31 links), focusing on animal models [35], while Europe prioritizes clinical applications [4]. This divergence hinders the creation of global therapeutic guidelines.

At the institutional level, Harvard University and Harvard Medical School lead in publication volume (n = 217), followed by France’s Université Paris Cité and APHP, and India’s L.V. Prasad Eye Institute. While this reflects the concentration of expertise in globally recognized hubs, it also underscores systemic biases: (1) 78% of Harvard’s output originates from NIH-funded projects (vs. <5% of LMIC institutions), (2) non-English publications from regions like Africa or Latin America are underrepresented in major indices, and (3) research agendas often prioritize high-impact interventions over local prevention needs (e.g., agricultural chemical burns in India). The prominence of L.V. Prasad Eye Institute, a leader in low-cost corneal therapies, demonstrates how inclusive funding models can diversify geographic contributions.

The journals also show a clear hierarchy, with “Burns” as the main journal in terms of the number of articles published (n = 306). Other important journals include “Cornea” and “Investigative Ophthalmology & Visual Science”, reflecting a significant focus on eye chemical burns.

The author analysis reveals that Reim M. and Sarfati E. are the authors with the highest number of publications (n = 35) [16], while Pfister R.R. is the author with the highest number of local citations (n = 577) and an h-index of 19, indicating the considerable impact of his work. The most influential authors defined critical axes in their research topics: Pfister R.R. [18] and Kuckelkorn [6] demonstrated that topical ascorbate reduces ulcerations by 78%, a finding replicated in 40% of post-2000 studies. While these breakthroughs emerged from independent clinical research, their citation impact may differ from industry-sponsored studies, which often prioritize patentable formulations over generic interventions like ascorbate, and Pellegrini and Rama stand out with their work on limbal cells, which not only generated 884 citations but also drove 20% of publications in tissue engineering (2010–2025) [25,26]. This highlights the need to contextualize bibliometric trends with funding sources, though our study focuses on foundational clinical evidence.

Simultaneously, the emerging topics reflect both technological innovation and pressing social realities, such as (1) nanotherapies (Tavakoli, 2021), (2) hydrogels with growth factors for burn regeneration [7], and (3) chemical assaults, a long-neglected issue where Mannan’s 2007 review [36] of 771 cases exposed critical patterns: Bangladesh’s disproportionate incidence (≥65% female victims) versus Jamaica’s high absolute numbers. Originally dismissed as ‘crimes of passion,’ these attacks are now recognized as gendered violence, catalyzing policy reforms (e.g., acid sales bans) and survivor-inclusive research paradigms.

Among the analyzed research, a variety of articles addressing different aspects of chemical burns are included. For example, articles are mentioned on pathophysiology and types of burns [1], management of chemical burns in emergency situations [5], treatment of eye burns [6], and general pathophysiology and treatment of chemical burns [4]. Epidemiological studies from different regions and specific topics, such as hand burns [38] and pediatric burns [37], are also included.

Among the topics addressed in scientific research on chemical burns, eye burns stand out significantly. It is the main MeSH term and the most represented category in scientific journals, with the highest presence among the top ten publications. Eye burns are, in fact, the primary focus of research in the field of chemical burns. Furthermore, they constitute a significant clinical issue in ophthalmology [6,39], underscoring the importance of this area of study.

### Limitations

This bibliometric analysis characterized global scientific papers on chemical burns, including thematic trends, citation networks, and collaboration patterns. However, several limitations must be acknowledged:

– Database bias:

Reliance on the Web of Science excluded regional journals and non-English publications, highlighting the contributions of the scientific journals with the greatest international projection in terms of visibility and impact and the publications of the researchers and institutions that participate in them, disproportionately omitting research from LMICs (Forjuoh, 2006) [2]. Only 5% of the analyzed studies addressed LMIC contexts, despite accounting for 93% of occupational burns in Bangladesh [40].

- Methodological constraints:

The bibliometric approach prioritized quantitative metrics (e.g., citation counts) over qualitative assessment of clinical impact. The animal model predominance (e.g., 65% of ophthalmological studies used rabbits [Wagoner, 1977] [27]) limited translational relevance to humans.

- Population gaps:

Pediatric populations were severely underrepresented (8.6% of studies [37]), despite a high incidence of household burns.

## 5. Conclusions

This study demonstrates that research on chemical burns has evolved significantly over the past eight decades, with two parallel trends. (a) Clinical focus: While *Burns* emerged as the most productive journal, its multidisciplinary scope (critical care, surgery, and dermatology) contrasted with the specialized ocular focus of the *Cornea* journal and ophthalmology journals (e.g., *IOVS* and *American Journal of Ophthalmology*), which dominated the MeSH term frequency (“Eye Burns”). This duality reflects the field’s progression from generalized burn management to targeted therapies for ocular injuries. (b) Translational gaps: Despite advances, some imbalances persist, with few studies addressing pediatric-specific needs, and LMIC-relevant research remains scarce. Future studies should prioritize mixed-method frameworks to evaluate therapy adoption across diverse healthcare contexts and interdisciplinary collaboration bridging technical innovations (e.g., bioengineering and immune modulation) with tangible survivor outcomes, especially for vulnerable groups. This transition from scientific productivity to societal impact remains the field’s defining challenge.

## Figures and Tables

**Table 1:** Impact of scientific production on chemical burns in the 10 most productive countries.

Countries	N Docs	% Docs	Dates	h-Index	Cit/Docs	Total Citations	Average Cit/Docs
USA	790	20.04	1973–2024	559	24.19	18,534	23.50
China	323	8.19	1981–2024	172	8.60	5849	18.10
UK	261	6.62	1986–2024	358	22.67	3856	14.80
Turkey	199	5.05	1973–2024	145	10.43	2758	13.90
India	175	4.44	1973–2024	160	8.44	3768	21.50
France	170	4.31	1973–2024	290	19.87	1930	11.40
Germany	154	3.91	1973–2024	302	18.19	2206	14.30
Japan	103	2.61	1973–2024	265	14.86	2623	25.50
Australia	91	2.31	2005–2024	241	21.74	1372	15.10
Italy	80	2.03	1985–2024	279	18.18	2737	34.20

Abbreviation: USA = United States of America; UK = United Kingdom; Docs = documents; Cit/docs = citations per document.

**Table 2:** Top 10 most productive journals on chemical burns.

Journal	n	%	JCR Category, Rank (Quartile)	IF	JCI	Start Year	Research Areas
Burns	306	7.76	Critical Care Medicine, 14/55, Q2 Dermatology, 25/94, Q2 Surgery, 43/292, Q1	3.2	0.91	1982	Development of regenerative therapies, management of chronic sequelae, and surgical innovations; analysis of safety, tolerability, and functional outcomes in ocular reconstruction
Cornea	132	3.35	Ophthalmology, 943/292 Q1	1.9	1.09	1998	Limbal and amniotic membrane transplants; analysis of prognostic factors, acute management, and chronic complications; epidemiological studies; and post-traumatic visual rehabilitation
Investigative Ophthalmology & Visual Science	99	2.51	Ophthalmology, 6/95, Q1	5.0	1.62	1977	Murine/rabbit models of alkali burns, strategies, immune modulation, and tissue repair; transcriptomic/proteomic analysis to identify therapeutic targets
Journal of Burn Care & Research	75	1.90	Critical Care Medicine, 36/55, Q3 Dermatology, 59/94, Q3 Surgery, 154/292, Q3	1.5	0.49	2006	Etiology and epidemiology of chemical burns and systemic and neurological complications; differential diagnosis, innovative therapies, pediatric and occupational injuries, and socioeconomic impacts
Journal of Pediatric Surgery	68	1.72	Pediatrics, 46/168, Q1 Surgery, 73/292, Q1	2.4	1.13	1971	Management of esophageal strictures due to caustic ingestion; experimental studies in animal models; and prognostic analysis of esophageal motility and post-burn gastroesophageal reflux
American Journal of Ophthalmology	67	1.70	Ophthalmology, 12/95, Q1	4.1	2.02	1946	Reconstructive techniques and comparison of efficacy in acute injuries; late complications and experimental studies on innovative topical therapies
Experimental Eye Research	62	1.57	Ophthalmology, 18/95, Q1	3.0	1.45	1972	Corneal repair mechanisms; innovative therapies to modulate signaling pathways and epithelial regeneration; and evaluation of biomaterials and growth factors to restore corneal transparency
Klinische Monatsblatter fur Augenheilkunde	48	1.22	Ophthalmology, 80/95, Q4	0.8	0.36	1972	Emergency management, late complications, experimental therapies, epidemiology, immunology, and visual rehabilitation
British Journal of Ophthalmology	43	1.09	Ophthalmology, 14/95, Q1	3.8	1.93	1952	Epithelial and amniotic transplants for limbal stem cell deficiency and ocular surface reconstruction; anti-inflammatory and antiangiogenic strategies; management of severe chemical injuries; and prognostic factors
Archives of Ophthalmology ^a^	39	0.98	Ophthalmology, 3/57, Q1	4.4	-	1947	Limbal cell and amniotic membrane transplants, ocular chemical burns, prevention of corneal ulceration, epithelial regeneration, and management of inflammation and neovascularization

The categories and impact factors are of the current journal. ^a^ Continues with *JAMA Ophthalmology* (from 2013), impact factor (2014 edition of Journal Citation Reports); Abbreviations: IF, impact factor (2023 edition of Journal Citation Reports); JCI, Journal Citation Indicator (2023 edition of Journal Citation Reports); JCR, Journal Citation Reports; n, number of documents; Q, quartile.

**Table 3:** Top 30 thematic categories of journals on chemical burns.

Rank	Category	N Docs	N Doc-Weighted Value	Rank	Category	N Docs	N Doc-Weighted Value
1	Ophthalmology	984	943	16	Respiratory System	84	36.92
2	Surgery	1185	719.83	17	Anesthesiology	40	35.58
3	Medicine, General and Internal	388	375.17	18	Pathology	52	35.17
4	Dermatology	547	260.67	19	Medicine, Legal	47	33.83
5	Pediatrics	325	218.83	20	Multidisciplinary Sciences	31	30
6	Gastroenterology and Hepatology	195	171.33	21	Biochemistry and Molecular Biology	48	25.42
7	Critical Care Medicine	430	151	22	Cardiac and Cardiovascular Systems	68	23.92
8	Emergency Medicine	168	135	23	Veterinary Sciences	22	16.5
9	Otorhinolaryngology	169	169	24	Orthopedics	29	16
10	Toxicology	139	105.5	25	Obstetrics and Gynecology	22	15.67
11	Public, Environmental, and Occupational Health	96	72.17	26	Cell Biology	30	14.52
12	Pharmacology and Pharmacy	75	52.17	27	Allergy	26	12.83
13	Medicine, Research, and Experimental	87	50.5	28	Immunology	20	10.17
14	Dentistry, Oral Surgery, and Medicine	55	50	29	Environmental Sciences	25	9.67
15	Radiology, Nuclear Medicine, and Medical Imaging	56	41.83	30	Oncology	16	9.6

**Table 4:** Top 10 most relevant authors on chemical burns in terms of production, citations, and impact.

Author	N Docs	h-Index	TCs	1st Publication	Topics Covered
Reim M. (1)	35	17	694	1973	Ischemia in chemical burns; innovative treatments for severe burns; acute erosions; enzyme activity; metabolic therapies; biomarkers; and surgical procedures (artificial epithelium) [13]
Sarfati E. (2)	35	16	836	1984	Management of digestive injuries following ingestion of corrosive substances; focus on adults with acute and chronic complications; and therapeutic strategies to prevent perforation and fibrosis [16]
Celerier M. (3)	32	10	493	1974	Management of caustic esophagitis, potassium permanganate gastric injuries, and surgical techniques for severe burns; strategies for esophageal reconstruction and gastric/colon bypass [17]
Pfister R. (4)	30	19	960	1971	Mechanisms of corneal repair and regeneration in caustic burns; collagenase inhibition and chemotactic agents to prevent ulceration and perforation; and antioxidant, anti-inflammatory, and modulatory therapies in experimental models [18]
Cattan P. (5)	23	15	687	2000	Surgical treatment of digestive injuries due to caustic ingestion (total esophagogastrectomy and other techniques); focusing on tissue necrosis, perforations, and systemic complications to prevent strictures and fibrosis [19]
Paterson C. (6)	22	15	800	1973	Ocular hypertensive response after chemical burns, such as changes in intraocular pressure and prostaglandin activity in aqueous humor; mechanisms of polymorphonuclear leukocyte infiltration and the effect of sodium citrate; and metalloproteinase inhibitor therapies to prevent corneal ulceration [20]
Kochhar R. (7)	21	11	791	1986	Prediction and management of esophageal and gastric strictures; evaluation of esophageal transit times, chronic complications, and enteral nutrition in severe injuries [21]
Basu S. (8)	20	13	1159	2011	Cultured autologous limbal epithelial transplants for recurrent ocular burns; penetrating keratoplasty after limbal transplantation; and combined techniques for chronic unilateral burns [22]
Dohlman Ch. (9)	20	15	792	1969	Use of bonded contact lenses for epithelial protection and regeneration in chemical burns; management of inflammatory glaucoma after ocular trauma; visual rehabilitation in eyes with phthisis; and development of keratoprostheses for severe cases of corneal damage [23]
Schrage N. (10)	19	12	403	1991	Ocular irrigation in chemical burns, impact of buffered solutions on corneal calcification, and use of amphoteric washes; diagnosis with microtrephine and acute management of chemical/thermal burns [24]

Abbreviations: n docs, number of documents; TCs, total citations. (1) Aachen University, the Department of Ophthalmology (Aachen, Germany), (2) Hôpital Saint-Louis, the Department of General, Endocrine, and Digestive Surgery (Paris, France), (3) Hôpital Saint-Louis, the Department of Surgery (Paris, France), (4) the Eye Research Foundation (Birmingham, United States), (5) Hôpital Saint-Louis, the Department of Digestive Surgery (Paris, France), (6) the University of Colorado Medical School, the Department of Ophthalmology (Denver, United States)/the University of Louisville, the Department of Ophthalmology and Visual Science (Louisville, United States), (7) the Postgraduate Institute of Medical Education and Research, the Department of Gastroenterology (Chandigarh, India)/Paras Hospital, the Department of Gastroenterology and Hepatology (Panchkula, India), (8) the L.V. Prasad Eye Institute (India), (9) Harvard Medical School, the Department of Ophthalmology/Massachusetts Eye and Ear (Boston, United States), and (10) the Aachen Centre of Technology Transfer in Ophthalmology/Aachen University, the Department of Ophthalmology (Aachen, Germany).

**Table 5:** Top 10 most locally cited documents on chemical burns.

Document	TCs	TCs/Year	NTCs	Document Type/Discipline	Research Topic
Pellegrini G. et al. Long-Term Restoration of Damaged Corneal Surfaces with Autologous Cultivated Corneal Epithelium. *Lancet* 1997, 349 (9057), 990–993, [25]	1100	37.93	26.94	Article/ general and internal medicine	Autologous transplant; stem cells; limbs; reconstruction and in vitro
Rama P., et al. Limbal Stem-Cell Therapy and Long-Term Corneal Regeneration. *N. Engl. J. Med.* 2010, 363 (2), 147–155. doi: 10.1056/NEJMoa0905955, [26]	884	55.25	23.66	Article/general and internal medicine	Autologous limbal stem cell transplants; eye and skin burns
Wagoner M.D. Chemical Injuries of the Eye: Current Concepts in Pathophysiology and Therapy. *Surv. Ophthalmol.* 1997, 41 (4), 275–313, [27]	384	13.24	9.40	Review/ophthalmology	Epidermal growth factor; conjunctival autograft transplantation; experimental alkali burns; epithelial basement membrane; and nasal mucosal transplantation
Oh J.Y., et al. The Anti-Inflammatory and Anti-Angiogenic Role of Mesenchymal Stem Cells in Corneal Wound Healing Following Chemical Injury. *Stem Cells* 2008, 26 (4), 1047–1055, [28]	312	17.33	13.05	Article/cell biology, biotechnology, applied microbiology, oncology, and hematology	Dendritic cells; cytokine expression; T cells; differentiation; thrombopondin-1; inhibition; neovascularization; and grafts
Meller D., et al. Amniotic Membrane Transplantation for Acute Chemical or Thermal Burns. *Ophthalmology* 2000, 107 (5), 980–989; discussion 990, [29]	309	11.88	8.23	Article/ophthalmology	Corneal surface reconstruction; ocular surface; limbal autograft; animal models; differentiation; and corneal perforation
Zargar S.A., et al. The Role of Fiberoptic Endoscopy in the Management of Corrosive Ingestion and Modified Endoscopic Classification of Burns. *Gastrointest. Endosc*. 1991, 37 (2), 165–169, [30]	297	8.49	14.32	Article/gastroenterology and hepatology	Ingestion of caustics; injuries in children; and esophageal and stomach injuries
Azuara-Blanco A., et al. Amniotic Membrane Transplantation for Ocular Surface Reconstruction. *Br. J. Ophthalmol*. 1999, 83 (4), 399–402, [31]	296	10.96	13.66	Article/ophthalmology	Innovative proposal for grafting techniques
Tsai R.J., et al. Human Allograft Limbal Transplantation for Corneal Surface Reconstruction. *Cornea* 1994, 13 (5), 389–400, [32]	296	9.25	14.5	Article/ophthalmology	Transplant technique for corneal reconstruction
Sangwan V.S., et al. Simple Limbal Epithelial Transplantation (SLET): A Novel Surgical Technique for the Treatment of Unilateral Limbal Stem Cell Deficiency. *Br. J. Ophthalmol*. 2012, 96 (7), 931–934, [33]	288	20.6	9.34	Article/ophthalmology	Transplantation; clinical results; culture; and cornea
Ma Y, et al. Reconstruction of Chemically Burned Rat Corneal Surface by Bone Marrow-Derived Human Mesenchymal Stem Cells. *Stem Cells* 2006, 24 (2), 315–321, [34]	282	14.1	12.05	Article/cell biology, biotechnology, applied microbiology, oncology, and hematology	Amniotic membrane transplant; ocular surface; stromal cells; differentiation; and epithelium

Abbreviations: NTCs, normalized total citations (total citations divided by expected number of citations for documents with the same publication year); TCs, total citations.

**Table 6:** Top 30 medical subject heading (MeSH) descriptors assigned to chemical burn papers across 3 time periods.

		1946–1974 (n = 333)		1975–1999 (n = 1495)		2000–2024 (n = 2101)		Overall (n = 3929)	
Rank	MeSH Heading	N Docs	%	N Docs	%	N Docs	%	N Docs	%
1	Eye Burns	70	21.02	381	25.48	707	33.65	1158	29.47
2	Esophageal Stenosis	77	23.12	337	22.54	269	12.8	683	17.38
3	Caustics	59	17.72	189	12.64	411	19.56	659	16.77
4	Esophagus	68	20.42	251	16.79	212	10.09	531	13.51
5	Cornea	42	12.61	151	10.1	250	11.9	443	11.28
6	Wound Healing	23	6.91	118	7.89	255	12.14	396	10.08
7	Sodium Hydroxide	28	8.41	121	8.09	214	10.19	363	9.24
8	Corneal Injuries	19	5.71	113	7.56	207	9.85	339	8.63
9	Alkalis	23	6.91	107	7.16	205	9.76	335	8.53
10	Corneal Diseases	0	0	42	2.81	184	8.76	226	5.75
11	Burns	53	15.92	63	4.21	86	4.09	202	5.14
12	Skin	20	6.01	71	4.75	102	4.85	193	4.91
13	Esophagoscopy	29	8.71	92	6.15	68	3.24	189	4.81
14	Stomach	29	8.71	103	6.89	55	2.62	187	4.76
15	Epithelium, Corneal	0	0	11	0.74	165	7.85	176	4.48
16	Visual Acuity	4	1.2	32	2.14	138	6.57	174	4.43
17	Postoperative Complications	18	5.41	96	6.42	60	2.86	174	4.43
18	Hydrofluoric Acid	3	0.9	77	5.15	84	4	164	4.17
19	Accidents, Occupational	7	2.1	79	5.28	75	3.57	161	4.1
20	Dilatation	20	6.01	79	5.28	58	2.76	157	4
21	Corneal Neovascularization	0	0	6	0.4	144	6.85	150	3.82
22	Limbus Corneae	0	0	14	0.94	133	6.33	147	3.74
23	Colon	13	3.9	74	4.95	49	2.33	136	3.46
24	Transplantation, Autologous	22	6.61	37	2.47	72	3.43	131	3.33
25	Skin Transplantation	10	3	48	3.21	71	3.38	129	3.28
26	Radiography	37	11.11	65	4.35	27	1.29	129	3.28
27	Therapeutic Irrigation	16	4.8	48	3.21	61	2.9	125	3.18
28	Esophagoplasty	14	4.2	81	5.42	28	1.33	123	3.13
29	Conjunctiva	11	3.3	56	3.75	53	2.52	120	3.05
30	Ophthalmic Solutions	0	0	27	1.81	91	4.33	118	3

**Table 7:** Research rankings of the 10 institutions with the most publications on chemical burns.

Affiliation	Country		Ranking (Percentile) ^a^				Line of Research
		N	O	R	I	S	
Harvard University/ Harvard Medical School/ Harvard University Medical Affiliates	USA	325	1	1	2	1	Topics on the management of chemical burns; burns involving various chemicals on the skin, eyes, and esophagus; and the various specific surgical and clinical techniques for their management (first publication: 1974)
Université Paris Cité	France	121	2	2	12	2	Treatment and management of chemical and caustic burns; diagnostic evaluation and experimental studies on wound healing and quality of life after internal injuries (first publication: 1974)
Assistance Publique Hôpitaux Paris (APHP)	France	118	1	1	10	1	Chemical burn treatment; case analysis and epidemiological studies on substance ingestion and its effects on health; and experimental models and evaluation of risk factors associated with chemical burns (first publication: 1974)
L. V. Prasad Eye Institute	India	91	59	46	73	49	Treatment and management of ocular chemical burns, including the use of conjunctival and amniotic membrane grafts; limbal stem cell transplantation; and specialty contact lenses (first publication: 1999)
University of California System	USA	78	1	1	7	1	Importance of prevention and timely treatment of chemical burns, understanding specific injuries and their health implications (first publication: 1973)
Chang Gung Memorial Hospital	China	68	14	19	25	11	They prioritize understanding chemical injuries, their management, and their impact on health, and developing new techniques and technologies to improve the treatment and reconstruction of affected tissues (first publication: 1988)
Hopital Universitaire Saint-Louis—APHP	France	68	-	-	-	-	They emphasize the need for a multidisciplinary approach to the management of severe chemical injuries, including early evaluation and reconstruction of the esophagus and gastrointestinal tract, and consideration of various risk factors (first publication: 1974)
Post Graduate Institute of Medical Education and Research, Chandigarh	India	61	-	-	-	-	Endoscopic techniques for managing chemical injuries in the respiratory and digestive tracts, also highlighting the need for long-term follow-up (first publication: 1979)
Egyptian Knowledge Bank	Egypt	59	-	-	-	-	The treatment and prevention of chemical injuries in children, management of esophageal strictures, management of stomach injuries, and epidemiological data on burns in children (first publication: 1976)
University of London	UK	56	23	14	41	25	Provides studies on various types of burns, including those involving chemical substances, iatrogenic injuries, oral and ocular injuries, and injuries caused by chemical ingestion (first publication: 1979)

^a^ Scimago research ranking, which refers to the volume, impact, and quality of the institution’s research output. Abbreviations: I, innovation ranking (based on the number of patent applications of the institution and the citations that its research output receives from patents); O, overall ranking; n, number of documents; R, research ranking; S, societal ranking (based on the number of pages of the institution’s website and the number of backlinks and mentions from social networks); UK, United kingdom.

## Data Availability

Data available in a publicly accessible repository; The original data presented in the study are openly available in Harvard Dataverse at: https://doi.org/10.7910/DVN/81102Q.

## Supplementary Materials

The following supporting information can be downloaded at https://www.mdpi.com/article/10.3390/ebj6030051/s1. Figure S1: Scientific production on chemical burns divided into 3 periods; Figure S2: Word cloud of term on chemical burns; Table S1: Main information on chemical burns publications; Table S2: Top 30 most cited countries on the scientific production on chemical burns; Table S3: Top countries in international collaboration; Table S4: Most relevant authors with highest number of local citations on chemical burns; Table S5: Top 60 Medical Subject Headings (MeSH) descriptors assigned to chemical burn papers across 3 time periods.
