# Supplementary material for: Scientific Production on Chemical Burns: A Bibliometric Analysis (1946–2024)
Source: Eur Burn J. 2025 Sep 9;6(3):51. doi: 10.3390/ebj6030051 (PMC12452322; doi:10.3390/ebj6030051)

“Scientific production on chemical burns: a bibliometric analysis (1946-2024)”

Table S1. Main information on chemical burns publications
Table S2. Top 30 most cited countries on the scientific production on chemical burns
Table S3. Top countries in international collaboration
Table S4. Most relevant authors with highest number of local citations on chemical burns
Table S5. Top 60 Medical Subject Headings (MeSH) descriptors assigned to chemical burn papers across 3 time periods.

Figure S1. Scientific production on chemical burns divided into 3 periods
Figure S2. Word cloud of term on chemical burns

Table S1. Main information on chemical burns publications

Description	Results
Timespan	1946-2025
Sources (Journals, Books, etc.)	757
Documents	3943
Annual Growth Rate %	0.65
Document Average Age	25.8
Average citations per doc	17.9
References	45139
**AUTHORS**	
Authors	11972
Authors of single-authored docs	440
**AUTHORS COLLABORATION**	
Single-authored docs	493
Co-Authors per Doc	4.04
International co-authorships %	6.214
**DOCUMENT TYPES**	
Article	3244
Letter	404
Note	120
Review	175

Table S2. Top 30 most cited countries on the scientific production on chemical burns

Country	Total citations	Average citation/documents
USA	18534	23.5
China	5849	18.1
UK	3856	14.8
India	3768	21.5
Turkey	2758	13.9
Italy	2737	34.2
Japan	2623	25.5
Germany	2206	14.3
France	1930	11.4
Korea	1763	22.9
Australia	1372	15.1
Israel	1139	17.5
Iran	964	21
Canada	870	18.5
Brazil	845	21.1
Spain	713	17.4
Sweden	566	28.3
Saudi Arabia	519	37.1
Belgium	409	17.8
Finland	333	27.8
Egypt	292	10.8
Switzerland	289	19.3
Czech Republic	276	23
Singapore	225	22.5
Mexico	213	16.4
South Africa	192	11.3
Greece	175	17.5
Denmark	160	14.5
Austria	122	7.6
Nigeria	110	6.9

Table S3. Top countries in international collaboration

Country	Destination Countries	%
USA	44	169
UK	35	59
France	25	42
Germany	16	18
Italy	15	21
Spain	14	25
China	13	31
Belgium	11	11
India	10	17
Austria	8	11
Canada	8	10
Brazil	7	16
Holland	7	7
Portugal	7	13
Swiss	7	9
Australia	6	11
Mexico	6	13
Argentina	5	8
Czech Republic	5	6

Abbreviations: UK, United Kingdom; USA, United States of America.

Table S4. Most relevant authors with highest number of local citations on chemical burns

Author	Local Citations
Pfister RR	577
Paterson CA	379
Reim M	370
Sarfati E	361
Kochhar R	302
Sangwan VS	257
Cattan P	250
Kuckelkorn R	244
Celerier M	240
Mehta SK	240
Mehta S	237
Zargar SA	237
Dohlman CH	222
Brown SI	209
Chirica M	197
Munoz-Bongrand N	186
Wagoner MD	185
Schrage NF	179
Nagi B	176

Table S5. Top 60 Medical Subject Headings (MeSH) descriptors assigned to chemical burn papers across 3 time periods.

		1946-1974 (n=333)		1975-1999 (n=1495)		2000-2024 (n=2101)		Overall (n=3929)	
**Rank**	**MeSH Heading**	**N docs**	**%**	**N docs**	**%**	**N docs**	**%**	**N docs**	**%**
1	Eye Burns	70	21.02	381	25.48	707	33.65	1158	29.47
2	Esophageal Stenosis	77	23.12	337	22.54	269	12.8	683	17.38
3	Caustics	59	17.72	189	12.64	411	19.56	659	16.77
4	Esophagus	68	20.42	251	16.79	212	10.09	531	13.51
5	Cornea	42	12.61	151	10.1	250	11.9	443	11.28
6	Wound Healing	23	6.91	118	7.89	255	12.14	396	10.08
7	Sodium Hydroxide	28	8.41	121	8.09	214	10.19	363	9.24
8	Corneal Injuries	19	5.71	113	7.56	207	9.85	339	8.63
9	Alkalies	23	6.91	107	7.16	205	9.76	335	8.53
10	Corneal Diseases	0	0	42	2.81	184	8.76	226	5.75
11	Burns	53	15.92	63	4.21	86	4.09	202	5.14
12	Skin	20	6.01	71	4.75	102	4.85	193	4.91
13	Esophagoscopy	29	8.71	92	6.15	68	3.24	189	4.81
14	Stomach	29	8.71	103	6.89	55	2.62	187	4.76
15	Epithelium, Corneal	0	0	11	0.74	165	7.85	176	4.48
16	Visual Acuity	4	1.2	32	2.14	138	6.57	174	4.43
17	Postoperative Complications	18	5.41	96	6.42	60	2.86	174	4.43
18	Hydrofluoric Acid	3	0.9	77	5.15	84	4	164	4.17
19	Accidents, Occupational	7	2.1	79	5.28	75	3.57	161	4.1
20	Dilatation	20	6.01	79	5.28	58	2.76	157	4
21	Corneal Neovascularization	0	0	6	0.4	144	6.85	150	3.82
22	Limbus Corneae	0	0	14	0.94	133	6.33	147	3.74

23	Colon	13	3.9	74	4.95	49	2.33	136	3.46
24	Transplantation, Autologous	22	6.61	37	2.47	72	3.43	131	3.33
25	Skin Transplantation	10	3	48	3.21	71	3.38	129	3.28
26	Radiography	37	11.11	65	4.35	27	1.29	129	3.28
27	Therapeutic Irrigation	16	4.8	48	3.21	61	2.9	125	3.18
28	Esophagoplasty	14	4.2	81	5.42	28	1.33	123	3.13
29	Conjunctiva	11	3.3	56	3.75	53	2.52	120	3.05
30	Ophthalmic Solutions	0	0	27	1.81	91	4.33	118	3
31	Eye Injuries	19	5.71	42	2.81	57	2.71	118	3
32	Administration, Topical	5	1.5	40	2.68	71	3.38	116	2.95
33	Anti-Bacterial Agents	17	5.11	44	2.94	52	2.48	113	2.88
34	Acids	16	4.8	41	2.74	49	2.33	106	2.7
35	Esophagitis	20	6.01	44	2.94	41	1.95	105	2.67
36	Corneal Ulcer	19	5.71	51	3.41	29	1.38	99	2.52
37	Cicatrix	9	2.7	48	3.21	40	1.9	97	2.47
38	Stem Cell Transplantation	0	0	9	0.6	88	4.19	97	2.47
39	Necrosis	19	5.71	35	2.34	42	2	96	2.44
40	Corneal Transplantation	10	3	27	1.81	57	2.71	94	2.39
41	Cells, Cultured	0	0	6	0.4	82	3.9	88	2.24
42	Burns, Electric	18	5.41	34	2.27	35	1.67	87	2.21
43	Surgical Flaps	0	0	38	2.54	49	2.33	87	2.21
44	Hydrochloric Acid	18	5.41	40	2.68	26	1.24	84	2.14
45	Suicide, Attempted	0	0	43	2.88	38	1.81	81	2.06
46	Amnion	0	0	2	0.13	79	3.76	81	2.06
47	Debridement	6	1.8	22	1.47	52	2.48	80	2.04
48	Hydrogen-Ion Concentration	14	4.2	26	1.74	38	1.81	78	1.99
49	Esophageal Neoplasms	14	4.2	48	3.21	15	0.71	77	1.96
50	Epithelium	14	4.2	54	3.61	8	0.38	76	1.93

51	Esophageal Diseases	19	5.71	33	2.21	24	1.14	76	1.93
52	Sulfuric Acids	10	3	36	2.41	29	1.38	75	1.91
53	Gastrectomy	10	3	38	2.54	27	1.29	75	1.91
54	Emergencies	3	0.9	49	3.28	22	1.05	74	1.88
55	Deglutition Disorders	4	1.2	29	1.94	41	1.95	74	1.88
56	Foreign Bodies	6	1.8	28	1.87	37	1.76	71	1.81
57	Occupational Diseases	12	3.6	39	2.61	20	0.95	71	1.81
58	Calcium Gluconate	1	0.3	32	2.14	36	1.71	69	1.76
59	Epithelial Cells	3	0.9	10	0.67	56	2.67	69	1.76
60	Facial Injuries	7	2.1	15	1	46	2.19	68	1.73

Figure S1. Scientific production on chemical burns divided into 3 periods

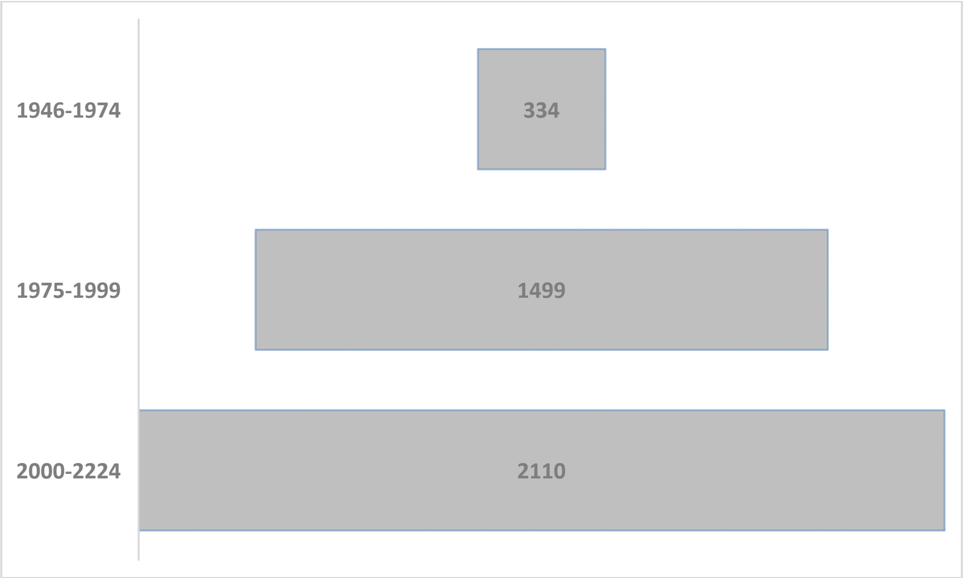

Figure S2. Word cloud of term on chemical burns

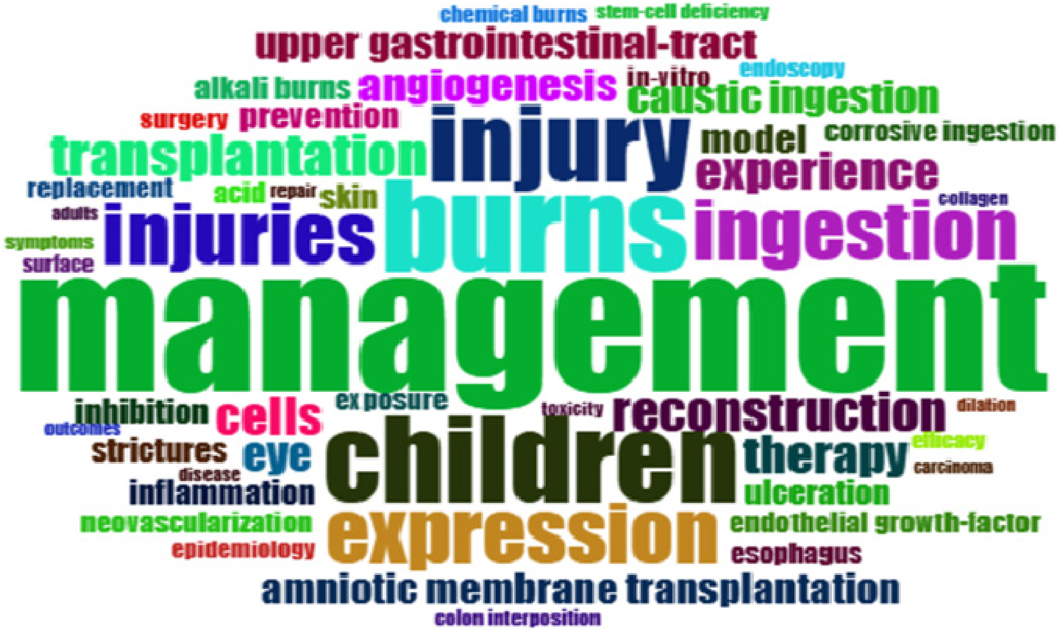

Supplement: Supplementary file 1 [file ebj-06-00051-s001.zip › ebj-3746569-supplementary.pdf]
